# Identification a rare chromosomal translocation 45,X, der(Y;15)(q11.2;q11.2) in an azoospermic patient using C-MoKa

**DOI:** 10.1186/s13039-026-00756-5

**Published:** 2026-03-27

**Authors:** Jia Deng, Min Liu, Xiaowen Liu, Xianghe Meng, Yuxia Yang, Yimin Wang, Liyuan Zhou, Aimin Deng, Jinhao Liu

**Affiliations:** 1 Hunan Provincial Key Laboratory of Regional Hereditary Birth Defects Prevention and Control, Reproductive Medicine Center, Changsha Hospital for Maternal & Child Health Care, Hunan Normal University, 416 Chengnan Road, Yuhua District, Changsha, 410007 China; 2 Yikon Genomics, Suzhou, 215125 China

**Keywords:** 45,X male, Azoospermia, Chromosome conformation based karyotyping, Short stature, Y chromosome translocation

## Abstract

**Background:**

The 45,X karyotype (X monosomy) is prevalent in females with Turner syndrome. However, in rare cases, it can also manifest in males. It typically results from an unbalanced translocation between the Y chromosome and an autosome, leading to the presence of the short arm of the Y chromosome (Yp) harboring the sex-determining region Y gene (SRY) and the concurrent deletion of the long arm of the Y chromosome (Yq) containing the azoospermia factor (AZF). This study aimed to identify the translocation of the SRY gene using chromosome conformation-based karyotyping (C-MoKa) and validate the results using FISH.

**Subject:**

A 32-year-old male, 155 cm in height, presented with normal sexual function but was unable to achieve clinical pregnancy after two years of unprotected intercourse. Three separate seminal fluid analyses indicated azoospermia. Chromosomal G-banding analysis suggested a 45,X karyotype, and CNV-seq identified a 15.16 Mb deletion in Yq11.21.

**Methods:**

Structural chromosomal variations were investigated using C-MoKa, a molecular karyotyping technique. According to the results, targeted probes were selected for validation via FISH.

**Results:**

C-MoKa identified a rare chromosomal translocation 45,X, der(Y;15)(q11.2;q11.2), whilst FISH analysis revealed that the patient harbored an abnormal derivative chromosome with positive signals for the CEPY/CEP15, XYpter and 15qter probes.

**Conclusions:**

In this study, the C-MoKa technique was used to identify a rare case of Y chromosome translocation involving chromosome 15 in a male with Turner syndrome, which was challenging to detect using conventional chromosomal G-banding and CNV-seq. C-MoKa is a rapid and accurate cytogenetic detection method that offers high-resolution detection of structural variations and improved breakpoint characterization, thereby overcoming the limitations of traditional karyotyping approaches.

**Supplementary Information:**

The online version contains supplementary material available at 10.1186/s13039-026-00756-5.

## Introduction

The 45,X karyotype (X monosomy) is commonly identified in individuals with Turner syndrome, generally resulting in a female phenotype. However, in rare cases, it can also be present in males. As is well documented, the majority of patients diagnosed as 45,X males are 45,X/46,XY mosaics, which can be detected via routine cytogenetic analysis [1]. Earlier studies have established that the 45,X male karyotype arises from an unbalanced translocation between the Y chromosome and an autosome, wherein the translocated segment onto the autosome is exclusively derived from the Y chromosome. Male differentiation in these patients may be attributed to the retention of Yp sequences, including SRY. Notably, the clinical phenotype of these patients can range from normal development to congenital malformations, depending on the location of Y chromosome breakpoints or autosomal breakpoints, and the size of deleted fragments. The translocation of a fragment of a Y chromosome to the short arm of the telocentric chromosome typically does not result in congenital malformations. Nevertheless, in some cases, dysspermatogenesis is associated with the deletion of Yq sequences, especially those related to AZF-containing regions.

The Y chromosome harbors genes essential for testicular development and function, such as the sex-determining region Y (SRY) located on Yp and the azoospermia factor (AZF) regions on Yq. It is an acrocentric chromosome with two pseudoautosomal regions (PAR1 and PAR2) at both terminal ends [2]. The Y chromosome is a haploid genome unique to males and lacks genes essential for survival. Due to its limited recombination capacity for DNA repair, major genomic structural alterations in the Y chromosome can be transmitted to subsequent generations [3], accounting for the survival of 45,X males. This article outlines a case of an azoospermic male with a 45,X karyotype, which was identified as a translocation between the Y chromosome and chromosome 15 using a new molecular karyotyping method and validated by FISH.

## Subject 

The patient, a 32-year-old male, was referred to our infertility center due to primary infertility. He had a height of 155 cm and a weight of 60 kg, with normal sexual function, and was unable to achieve clinical pregnancy after 2 years of unprotected intercourse. The patient exhibited a male phenotype with normal primary sexual characteristics, including normal penile growth, but had sparse facial and pubic hair. He was an only child and reported no family history of genetic disorders or consanguineous relationships. According to the patient and his parents, his growth and development were comparable to age-matched peers, except for his shorter stature. Laboratory examinations revealed azoospermia in three separate seminal fluid analyses. Scrotal ultrasonography revealed bilaterally small testes, each measuring approximately 5 mL. The internal diameter of the left spermatic vein was 1.8 mm, which increased to 2.3 mm during the Valsalva maneuver. Color Doppler flow imaging (CDFI) detected no retrograde blood flow, and the right spermatic vein appeared normal. Meanwhile, hormonal analysis showed follicle-stimulating hormone (FSH) levels of 15.1 IU/L, luteinizing hormone (LH) levels of 6.11 IU/L, total testosterone (T) levels of 8.34 nmol/L, and inhibin B (INHB) levels of 15.33 pg/mL.

## Methods

### 1. Karyotyping

Cytogenetic analysis was performed on metaphase chromosomes from peripheral lymphocytes, which were trypsin-digested and stained using standard G-banding protocols to achieve 550-band resolution. Following standard procedures, fifty metaphase spreads were randomly examined, with five suitable ones selected for karyotyping. The resulting karyotypes were verified against the latest International System for Human Cytogenomic Nomenclature 2024 (ISCN 2024) guidelines [4].

### 2. DNA extraction

After obtaining informed consent, peripheral venous blood was collected from the patient with EDTA as the anticoagulant. Genomic DNA was extracted using the QIAamp DNA Mini Kit (QIAGEN, Germany) in accordance with the manufacturer’s instructions. DNA quality was evaluated to confirm a concentration of over 5 ng/µL and an OD_260_/OD_280_ ratio ranging from 1.8 to 2.0, as measured by a Nanodrop 1 C ultraviolet spectrophotometer (Thermo Fisher Scientific, USA).

### 3. Y chromosome microdeletion

Y chromosome-specific sequences were detected using the short tandem repeat (STR) method. A total of 15 sequence-tagged sites (STS) in the azoospermic factor (AZF) regions were evaluated, including six core STS: AZFa region (SY84, SY86), AZFb region (SY134, SY127), and AZFc region (SY254, SY255). Moreover, eight extended STS [AZFa region (SY82, SY88, SY1064, SY1065), AZFb region (SY105, SY121), and AZFb/c region (SY153, SY1192)], one heterochromosomal marker SY160, and the SRY gene were assessed. In addition, microdeletion analysis of the azoospermic factor (AZF) regions and the SRY gene was carried out using multiplex PCR. Finally, the amplicons were subjected to capillary electrophoresis on an ABI 3500Dx Genetic Analyzer. Data analysis was performed using GeneMapper software.

### 4. Copy number variant-sequencing (CNV-seq)

High-throughput sequencing technology was applied for genomic DNA analysis, integrating short tandem repeat (STR) analysis with methylation-specific PCR (MS-PCR) to detect aneuploidy and triploidy across all 23 chromosome pairs, as well as deletions or duplications exceeding 100 kb. Briefly, a DNA library was constructed according to next-generation sequencing (NGS) protocols. After end repair, an A-tail and an adapter were added, followed by PCR amplification. The library was subjected to qualitative and quantitative assessments using an Agilent 2100 Bioanalyzer and quantitative PCR (qPCR). The library DNA was sequenced at 0.3× low coverage on the NGS platform. The Population-Scale Copy Number Variation (PSCC) algorithm [5] was employed for statistical analysis of the data. Chromosomal breakpoint variations were identified using the t-test, and results with a P-value below the set threshold (0.001) were considered statistically significant for further evaluation. Copy number losses were inferred when the copy ratio was less than 0.7, whereas duplications were defined as having a relative ratio greater than 1.3.

### 5. C-MoKa detection

C-Moka was performed as described in a previous study [6]. Briefly, mononuclear cells were isolated from 2 mL of anticoagulated blood and fixed with 2% formaldehyde. The cells were then lysed, followed by chromosomal digestion, DNA labeling, and ligation. After reversing formaldehyde crosslinks, DNA fragmentation and library preparation were performed using reagents with catalog numbers KT110700324 and KT100804048 (Yikon Genomics Ltd). The DNA products were subsequently amplified, and the libraries were sequenced using 30 million read pairs on a DNBSEQ-T7 platform (MGI).

Raw sequencing data were initially processed using Juicer v1.6 to generate an initial spatial contact matrix with a window size of 1 MB. Next, the matrix was normalized against the reference system to obtain a corrected spatial contact matrix. For the identification of structural variations, putative positive signals were inspected to confirm that the Observed/Expected ratios exceeded 2.

### 6. Fluorescence In Situ Hybridization (FISH) validation

Three probe combinations were hybridized to metaphase chromosomes derived from cultured peripheral blood lymphocytes. Group 1 consisted of XYpter (green) and XYqter (red) probes, while Group 2 comprised 15qter (red) probe, and Group 3 encompassed CEPY (red) and CEP15 (green) probes (Abbott Molecular Vysis, Illinois, USA). A total of 30 metaphase cells were observed under a fluorescence microscope according to the manufacturer’s protocol for the FISH detection reagents.

## Results

### 1. Karyotyping

**Fig. 1:**
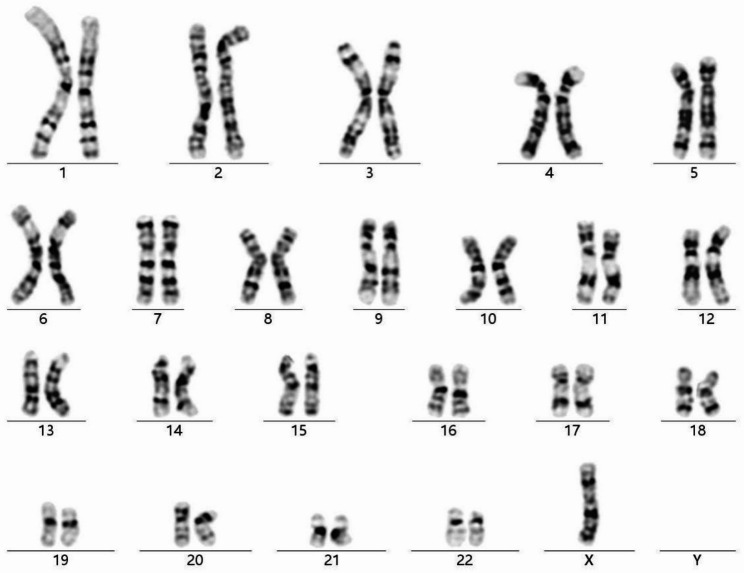
Karyogram of the 45,X male patient

G-banding analysis revealed a 45,X karyotype in all 50 metaphase spreads. Notably, the analysis was extended to 200 metaphase divisions to exclude the possibility of low-level mosaicism, and all of these divisions exhibited a 45,X karyotype (Fig. 1).

### 2. Y chromosome microdeletions

All 15 STS sites, including the sY160 site in Yq12, were deleted. In contrast, the SRY site yielded a positive result.

### 3. CNV-seq

**Fig. 2:**
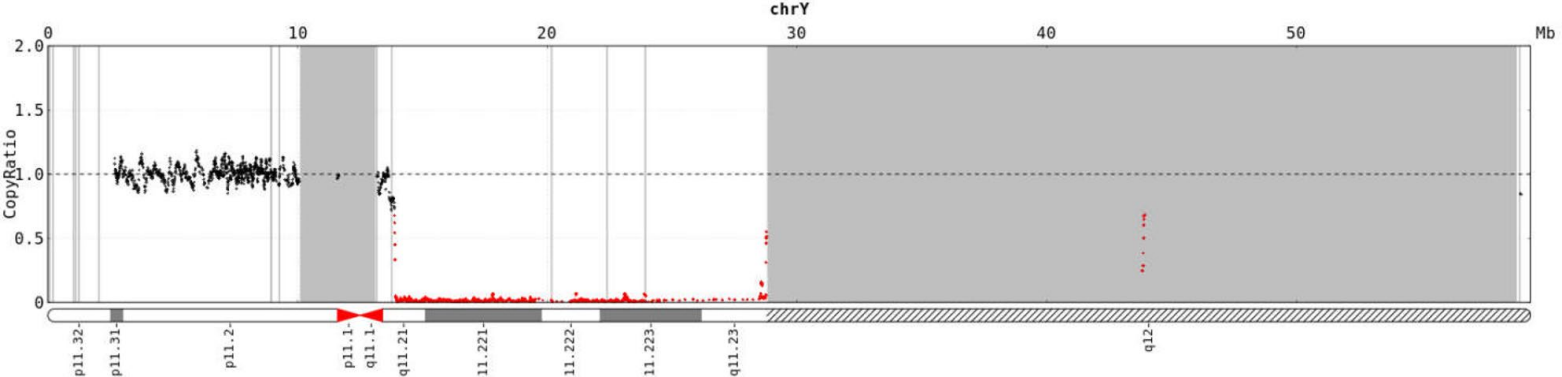
CNV-seq results of the patient. The patient exhibited a deletion in the Yq region, with the red bar indicating the deleted area

CNV sequencing identified a deletion at [GRCh37]Yq11.21-q12(chrY:13657068_28819361), corresponding to a loss of approximately 15.16 Mb in the Yq11.21-q12 region (Fig. 2).

### 4.C-MoKa detection

**Fig. 3:**
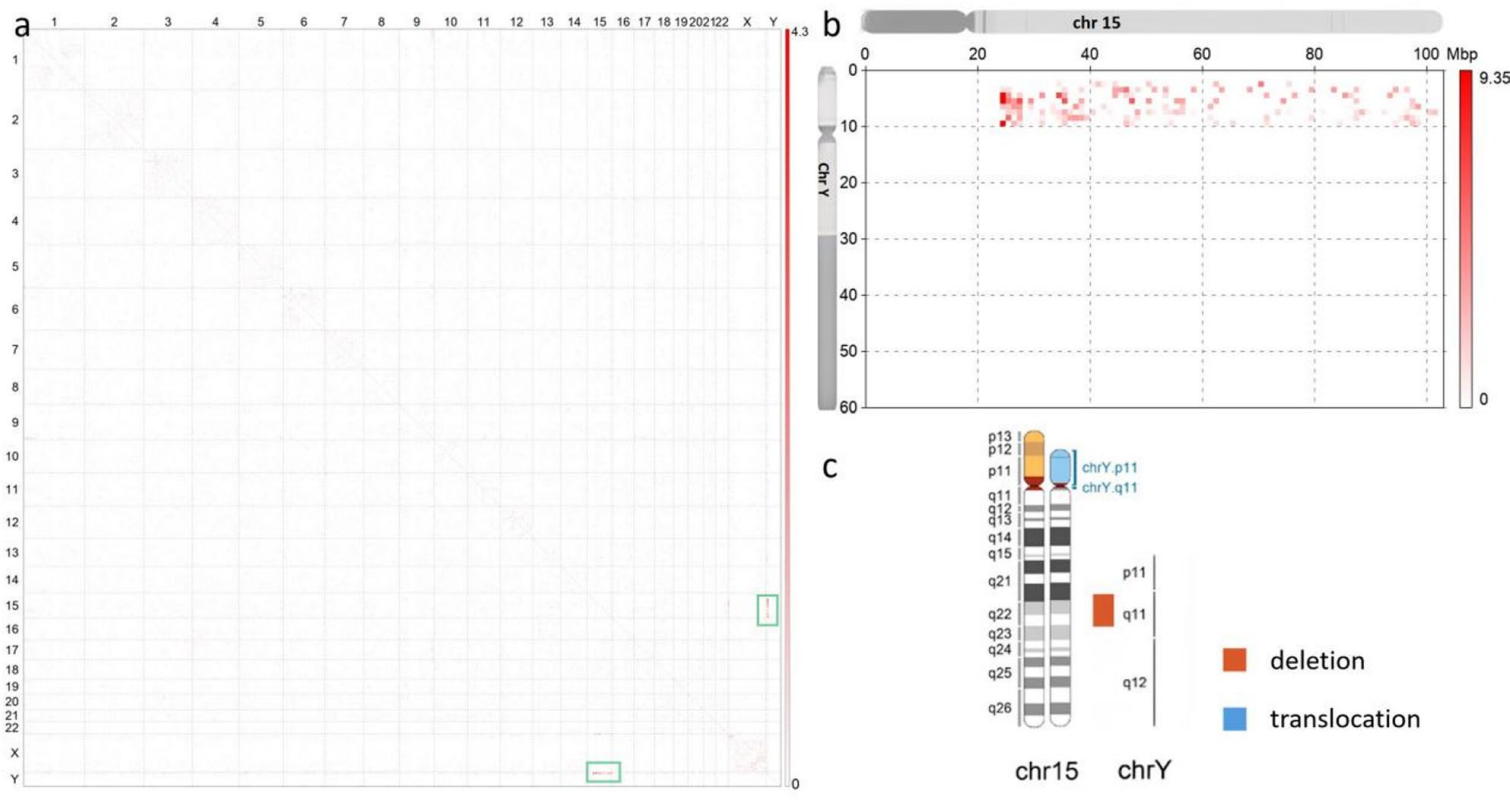
C-MoKa results of the patient. **a**: Contact map displaying the translocation event der(Y;15)(q11.2;q11.2), as indicated by intensified interaction signals between chr15 and chrY in the rectangles. **b**: Magnified view of the interaction between chr15 and chrY. **c**: Schematic representation of the chromosomal structural variation: der(Y;15)(q11.2;q11.2)

**Fig. 4:**
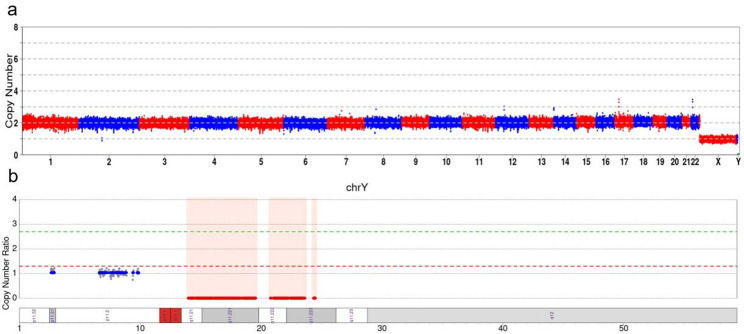
CNV analysis results via C-MoKa. **a**: No CNV alterations were identified in chromosomes other than chrY. **b**: Magnified view of chrY, revealing a deletion at Yq11.21-q11.223(chrY:13,980,001_24,520,000)del

To locate the SRY gene, C-MoKa detection was performed. A translocation event involving der(Y;15)(q11.2; q11.2) was visualized in the special contact map, as illustrated in Fig. 3. Moreover, the deletion event [GRCh37]Yq11.21-q11.223(chrY:13,980,001_24,520,000) was detected, in line with the results of CNV-seq. Lastly, an approximately 10.54 Mb deletion was identified in the Y chromosome (Fig. 4).

### 5. FISH validation

**Fig. 5:**
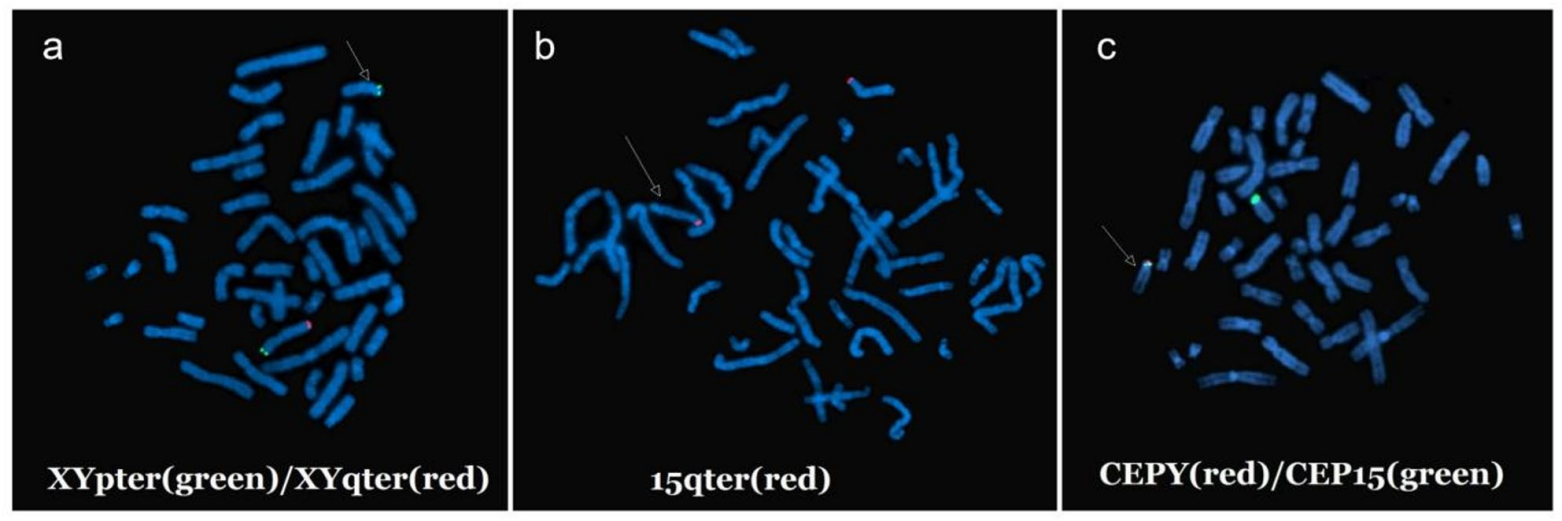
FISH images of metaphase cells from the patient, detected using different probes

In FISH Group 1, hybridization with XYpter/XYqter probes combined with DAPI staining revealed the presence of a normal X chromosome: the XYqter probe showed a red signal on the long arm, and the XYpter probe showed a green signal on the short arm. Another chromosome exhibited only a green signal from the XYpter probe (Fig. 5a).

In FISH assay Group 2, two chromosomes showed a red signal from the 15qter probe (Fig. 5b).

In FISH Group 3, one chromosome showed a green signal from the CEP15 probe, while another chromosome showed both a red signal from the CEPY probe and a green signal from the CEP15 probe (Fig. 5c).

These results collectively indicated that the patient harbored an abnormal derivative chromosome with positive signals for the CEPY, CEP15, XYpter, and 15qter probes. Notably, the CEP15 probe signal on this derivative chromosome was weaker than that on the normal chromosome 15.

## Discussion

This study reports a rare case of Y-15 chromosome translocation in a male patient with Turner syndrome, which is difficult to detect using conventional G-banding. Of note, karyotype analysis has several limitations: it has low resolution, as it can only detect fragments larger than 5–10 Mb; it cannot accurately identify the breakpoints of structural variations; and the differences between the dark and light bands of chromosomes are not evident. All these limitations collectively increase the risk of missed diagnoses. Even in conjunction with CNV-seq and other technologies, the presence of a 45,X/46,XY mosaicism cannot be ruled out. To avoid missed detection of the Y chromosome material, the use of C-Moka technology or FISH for confirmation is essential. Indeed, C-MoKa enables genome-wide detection of chromosomal structural abnormalities with higher resolution than G-banding. More specifically, it offers a resolution of 1 Mb for fragments and 100 kb for breakpoints [6]. Compared to CNV-seq, C-MoKa can not only detect balanced structural variations but also determine the orientation of duplications and insertions.

The C-MoKa method is based on Hi-C technology. Herein, prior to adopting C-MoKa, Optical Genome Mapping (OGM) was used to identify structural variations but obtained negative results (see Supplementary Figs. 1 and 2). OGM and long-read sequencing methods (e.g., Nanopore sequencing) cannot detect structural variation breakpoints and exhibit limited resolution in centromeric regions and repetitive sequences where translocations frequently occur. For instance, neither OGM nor Nanopore sequencing can detect Robertsonian translocations, a limitation not applicable to C-MoKa. Additionally, from a clinical perspective, the costs of both OGM and Nanopore sequencing remain prohibitively high. In comparison, C-MoKa requires only one-tenth to one-third the cost of long-read Sequencing, OGM, or solution capture methods. Moreover, C-MoKa can resolve complex structural variations with a relatively modest sequencing depth (30 million reads). As a novel molecular technique, it has demonstrated significant promise and advantages in detecting chromosomal structural abnormalities, CNVs, and uniparental disomy(UPD). It is a valuable tool for complex and rare cases of chromosomal abnormalities, positioning C-MoKa as a key diagnostic tool.

Some researchers argue that the 45,X karyotype in males does not exist and that, instead, the Y chromosome containing the SRY region undergoes an unbalanced translocation with other chromosomes [7]. In the present case, unbalanced translocation between the patient’s Y chromosome and chromosome 15 resulted in the complete deletion of the AZF. This deletion ultimately culminating in congenital testicular dysplasia, which further progressed to hypergonadotropic hypogonadism, characterized by reduced testosterone secretion, impaired spermatogenesis, and elevated FSH and LH levels.

Reported translocations involve almost every chromosome, with the majority being proximal centromeric translocations in group D (chromosomes 13, 14, and 15) and group G (chromosomes 21 and 22), especially between the Y chromosome and chromosome 15 [8]. This phenomenon may be attributed to sequence homology between the heterochromatin regions of 15p and Yq [9]. Additionally, t(Y;15) typically results in an unbalanced translocation, where the majority of breakpoints are located on 15p, specifically within the 15p11-p13 region and in the heterochromatic region of Yq12. In the present study, the patient harbored an unbalanced translocation, 45,X, der(Y;15)(q11.2;q11.2), as validated by C-MoKa. However, the possibility that the precise breakpoint lies within the 15p11-p13 region cannot be excluded. This is because this region consists predominantly of heterochromatin, which cannot be detected using NGS or other molecular genetic techniques. Given the patient’s sequencing data volume (30 million 150-bp paired-end reads), the resolution for structural variant (SV) breakpoints and CNV breakpoints was approximately 100 kb and 20 kb, respectively. However, because the 15q11.2 region is located near the centromere, determining the precise CNV breakpoint was challenging. No deletion was detected in the 15q11.2 region, as supported by the local CNV profile of chromosome 15 (see Supplementary Fig. 3). More importantly, the patient did not manifest clinical features of Burnside-Butler syndrome or Prader-Willi syndrome. FISH analysis revealed the presence of dicentric fragments on derivative chromosomes originating from chr15 and chrY, implying that the breakpoints are located at 15p11-p13.

Additionally, the two broken chromosomes, each containing centromeric regions, fused to create a dicentric aberration. It is worthwhile acknowledging that dicentric chromosomes resulting from translocations between chromosomes Y and 15 are rare [10]. As one of the centromeres is inactive or nonfunctional, the dicentric chromosome separated during cell division may segregate as a monocentric chromosome [11, 12]. The 15p and Yq acentric fragments are prone to loss during subsequent cell divisions. Consequently, only 45 chromosomes remained. Conventional G-banding cytogenetic analysis cannot precisely distinguish the derived chromosome 15, which contains a small segment of Yp, from normal chromosomes.

Adult males with a 45,X karyotype are generally diagnosed after marriage, when they seek medical treatment for infertility. Some patients may present with intellectual disability, short stature, or other symptoms. These symptoms may arise from the translocation of Y chromosome with other chromosomes, leading to the inactivation of gene expression on the translocated chromosome. The severity of clinical features in these patients is closely related to the type of Y chromosome translocation and the location of the translocation breakpoints [14–16]. Specifically, for 45,X males with translocations between chromosome 15 and the Y chromosome, the location of chromosome breakpoints and clinical phenotypes are summarized in Table 1.

**Table 1:** Summary of cases of 45 X males with Y;15 translocations

Case	Authors (yr) (ref.)	Chromosomal karyotype	Age at diagnosis	Clinical phenotype	Analytical method
1	Subrt I et al., [13]	45,X, t(Y;15)(Yqter→Yp11::15q11→15qter)	16 m	Four males from four consecutive generations of a pedigree harbored 45,X, t(Y;15) translocation and a normal phenotype.	Karyotype
2	Schempp W et al., [14] and Gal A et al., [10] Mancini Aet al, [15] report the same case	45,X, der(15)(Ypter→q11.21::15p11.2→qter)	19 y	Azoospermia, short stature (154 cm), normal mental development, and no deformity.	Karyotype, Y DNA-hybridization, FISH
3	Disteche CM et al., [16]	45,X, t(Y:15)carry the entire short arm), the centromere, and the proximal long arm of the Y chromosome	17 m	Mental retardation, growth retardation, microcephaly, spastic quadriplegia, undescended testes, and no features of Prader-Willi syndrome.	Karyotype, in situ hybridization
4	Farah SB et al., [17]	45,X(Y:15)	6 y	Developmental delay, mental retardation, strabismus, with male external genitalia and normal penis and scrotum.	Karyotype, in situ hybridization
5	White LM et al., [18]	45,X, dic(Y;15)(q11.23;p11.1)	fetus	The karyotype of the fetus was identical to that of the father, with no abnormal phenotype observed.	Karyotype, FISH Microsatellite analysis
6	Wimmer R et al., [19]	45,X, psu dic(15;Y)(15qter→15p11.2::Yq12→Ypter)	11 y	All male family members displayed a 45,X, psu dic(15; Y) karyotype, including the proband’s father and grandfather.	Karyotype, FISH
7	Lin S et al., [20]	45,X, der(15)(?::p11.2→qter)dn.ish psu dic(Y;15)(q12;p11.2) (D15Z1+,SNRPN+,PML+;SRY+,DYZ3+,DYZ1+)	33y	Normal intelligence, growth and development, testicular size, and sex hormone level, but infertility. The patient developed severe oligoasthenospermia due to partial AZFc (sY254) deletion.	Karyotype, FISH, Multiplex PCR
8	Yıldırım MS et al., [21]	45,X, t(15;Y) (q11;q12)	1y	A 1-year-old boy with no apparent dysmorphic features, disease, or complaints. His father had a t(15;20)(q11;p13)karyotype.	Karyotype, FISH
9	Qin Set al., [22]	45,X, dic(Y;15)(q11;p11).ish dic(Y;15)(SRY+,DYZ3+;D15Z1+,PML+)	27y	160 cm and small testes. Laboratory tests revealed normal testosterone level and high follicle-stimulating hormone level. The patient suffered from azoospermia due to deletion of AZFa + b+c loci and had a 45,X karyotype.	Karyotype, FISH, Multiplex PCR, CMA

Herein, the patient exhibited a short stature, a common phenotype in 45, X males [14, 22]. Previous research has identified deletion of the short stature homeobox-containing gene (*SHOX*) in PAR1, located on the short arm of the Y chromosome, as a critical determinant of short stature in patients with Turner syndrome [23]. Notably, the *SHOX* gene is located at Yp11.32, near the end of the short arm, and has a homologous gene on the X chromosome. Fukami et al. [24] described that mutations, deletions, and CNVs in the *SHOX* gene are significant genetic factors leading to *SHOX* haploinsufficiency, thereby impacting bone growth. In this study, the patient was not evaluated for the presence of the *SHOX* gene. A case report by Mareri et al. outlined a 14-year-old male diagnosed with growth hormone deficiency and delayed puberty, who harbored a 45,X, dic(Y;21)(q11;p11).ish dic (Y;21)(SRY+, SHOX+, DYZ3+, D21Z1+) karyotype. Despite the absence of Yp, the patient achieved the expected height after 6 months and 24 months of treatment with human chorionic gonadotropin (hCG) and growth hormone (GH) [25]. Earlier studies have concluded that boys with 45,X/46,X, idic (Yp) karyotypes and short stature may benefit from early GH therapy at pharmacologic doses [26].

At present, key questions remain regarding whether GH therapy can achieve the expected outcomes for 45,X male patients. It is also vital to identify the chromosome translocation with the Y chromosome and to locate breakpoints and deletion sequences of both chromosomes to evaluate other accompanying clinical phenotypes. Addressing these issues requires close collaboration between clinicians and laboratory specialists to deepen the understanding of the association between breakpoint deletions and phenotypic outcomes in patients with Y chromosome and autosomal translocations. This collaboration will provide a robust theoretical basis for prenatal diagnosis and early clinical intervention. In this study, C-MoKa, a novel molecular diagnostic technique, was successfully applied to identify the chromosomal abnormality 45,X, der(Y;15)(q11.2;q11.2), which was validated by FISH. Owing to its high efficiency, resolution, and accuracy, C-MoKa is anticipated to emerge as a reliable tool for detecting chromosomal structural variations.

## Conclusion

This study reports a rare case of a 32-year-old male patient with Turner syndrome who presented with azoospermia. Using the novel C-MoKa technique, a rare chromosomal translocation, 45,X, der(Y;15)(q11.2;q11.2), was identified and subsequently validated by FISH. These results indicate that C-MoKa serves as a rapid and accurate cytogenetic detection method, offering high-resolution identification of structural chromosomal variations.

In 45,X males with Y;15 translocations, primary infertility and short stature constitute a prevalent clinical phenotype. The novel molecular karyotyping technique has the potential to deepen our understanding of the relationship between breakpoints/deletions and phenotypic outcomes in patients with Y chromosome and autosomal translocations. This advancement provides a robust theoretical basis for prenatal diagnosis and early clinical intervention in such patients.

## Supplementary Information

Below is the link to the electronic supplementary material.

Supplementary Material 1

## Data Availability

No datasets were generated or analysed during the current study.
