# Supplementary material for: Identification a rare chromosomal translocation 45,X, der(Y;15)(q11.2;q11.2) in an azoospermic patient using C-MoKa
Source: Mol Cytogenet. 2026 Mar 27;19:18. doi: 10.1186/s13039-026-00756-5 (PMC13147550; doi:10.1186/s13039-026-00756-5)
Supplement: Supplementary file 1 — Supplementary Material 1 [file 13039_2026_756_MOESM1_ESM.pptx]

## Slide 1
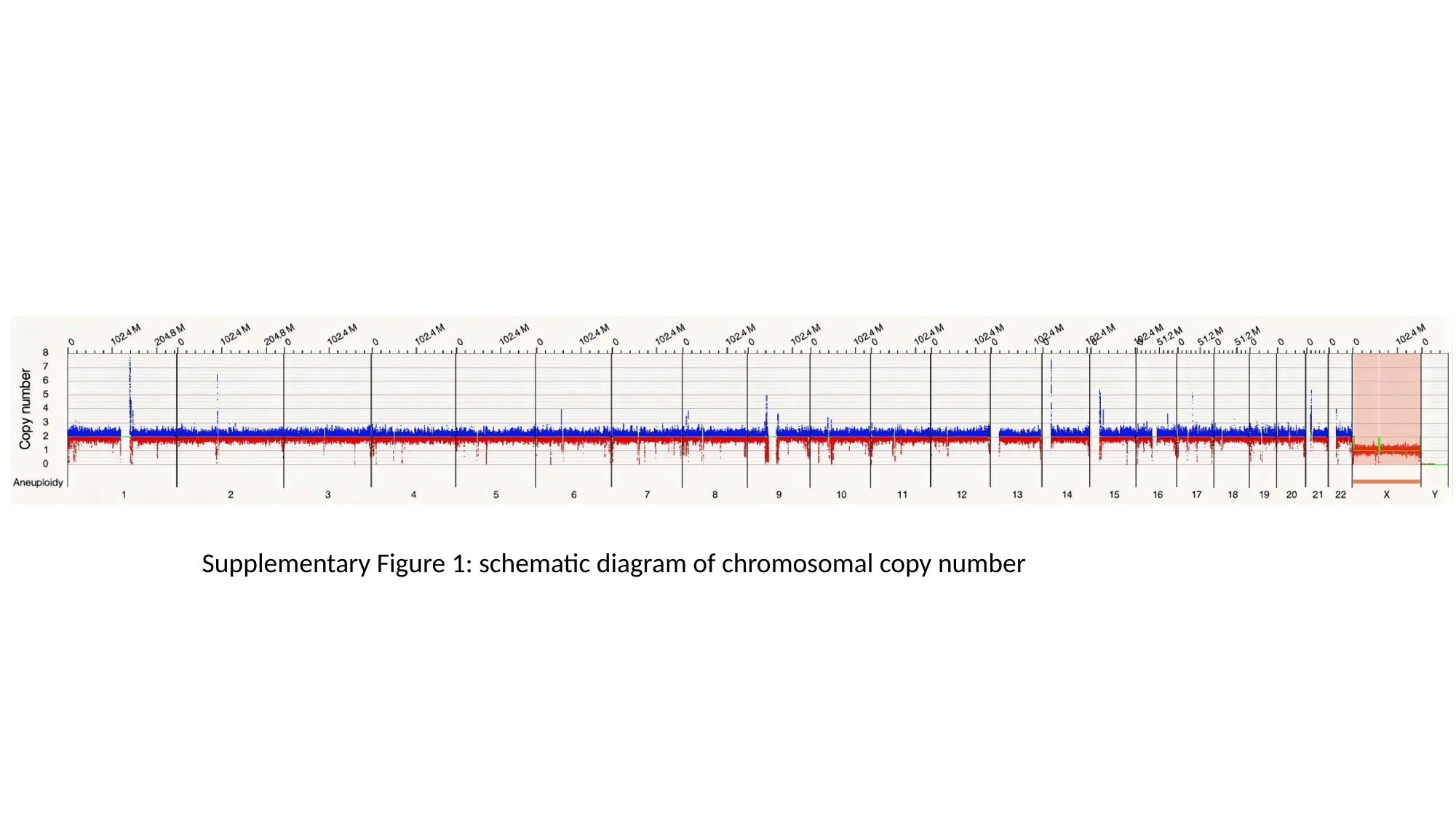

Supplementary Figure 1: schematic diagram of chromosomal copy number

## Slide 2
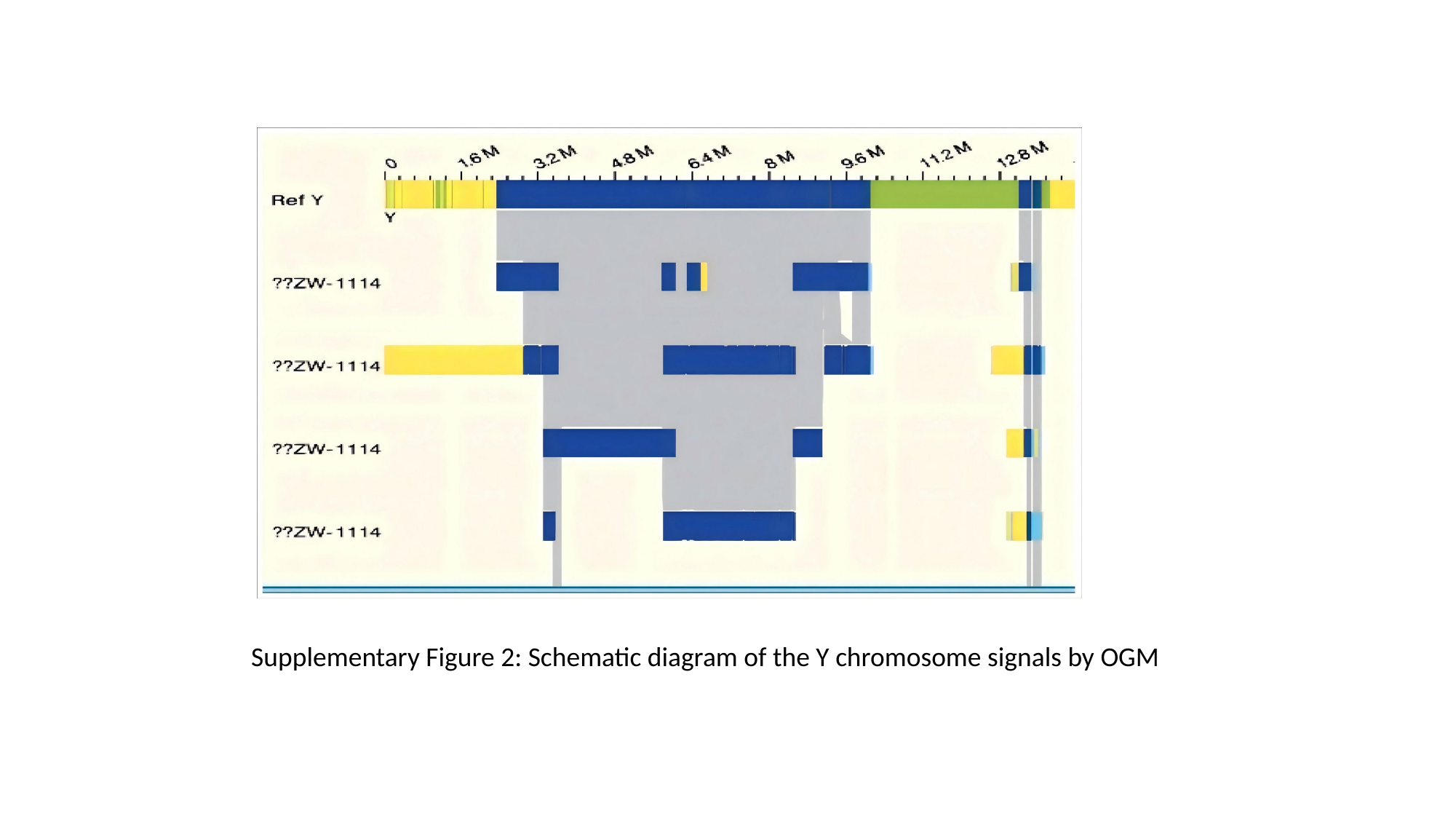

Supplementary Figure 2: Schematic diagram of the Y chromosome signals by OGM

## Slide 3
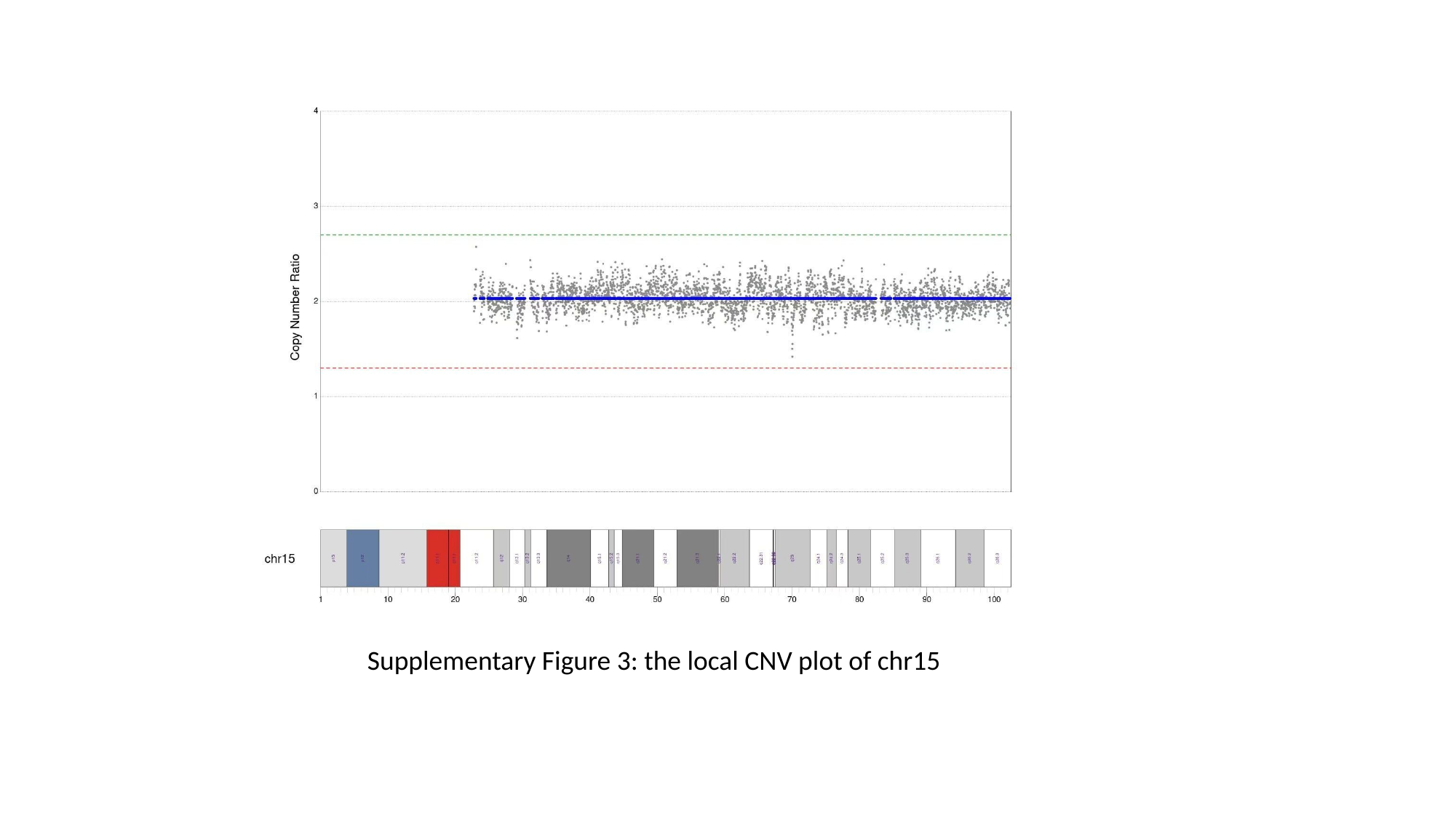

Supplementary Figure 3: the local CNV plot of chr15
